# Cost analysis of options for management of African Animal Trypanosomiasis using interventions targeted at cattle in Tororo District; south-eastern Uganda

**DOI:** 10.1186/s13071-015-0998-8

**Published:** 2015-07-22

**Authors:** Dennis Muhanguzi, Walter O. Okello, John D. Kabasa, Charles Waiswa, Susan C. Welburn, Alexandra P.M. Shaw

**Affiliations:** Department of Biomolecular and Biolaboratory Sciences, School of Biosecurity, Biotechnical and Laboratory Sciences, College of Veterinary Medicine Animal Resources and Biosecurity, Makerere University, P.O. Box 7062, Kampala, Uganda; Division of Infection and Pathway Medicine, Centre for Infectious Diseases, School of Biomedical Sciences, College of Medicine and Veterinary Medicine, The University of Edinburgh, Chancellor’s Building, 49 Little France Crescent, Edinburgh, EH16 4SB UK; Department of Biosecurity, Ecosystems & Veterinary Public Health, School of Biosecurity, Biotechnical and Laboratory Sciences, College of Veterinary Medicine Animal Resources and Biosecurity, Makerere University, P.O. Box 7062, Kampala, Uganda; Department of Pharmacy, Clinical and Comparative Medicine, School of Veterinary Medicine and Animal Resources, College of Veterinary Medicine Animal Resources and Biosecurity, Makerere University, P.O. Box 7062, Kampala, Uganda; Avia-GIS, Risschotlei 33, B-2980 Zoersel, Belgium

**Keywords:** African animal trypanosomiasis, Cattle, Cost, Human African trypanosomiasis, Restricted insecticide application protocol, Trypanocides, Tsetse control, Tick-borne diseases, *Trypanosoma*, *Theileria parva*

## Abstract

**Background:**

Tsetse-transmitted African trypanosomes cause both nagana (African animal Trypanosomiasis-AAT) and sleeping sickness (human African Trypanosomiasis - HAT) across Sub-Saharan Africa. Vector control and chemotherapy are the contemporary methods of tsetse and trypanosomiasis control in this region. In most African countries, including Uganda, veterinary services have been decentralised and privatised. As a result, livestock keepers meet the costs of most of these services. To be sustainable, AAT control programs need to tailor tsetse control to the inelastic budgets of resource-poor small scale farmers. To guide the process of tsetse and AAT control toolkit selection, that now, more than ever before, needs to optimise resources, the costs of different tsetse and trypanosomiasis control options need to be determined.

**Methods:**

A detailed costing of the restricted application protocol (RAP) for African trypanosomiasis control in Tororo District was undertaken between June 2012 and December 2013. A full cost calculation approach was used; including all overheads, delivery costs, depreciation and netting out transfer payments to calculate the economic (societal) cost of the intervention. Calculations were undertaken in Microsoft Excel™ without incorporating probabilistic elements.

**Results:**

The cost of delivering RAP to the project was US$ 6.89 per animal per year while that of 4 doses of a curative trypanocide per animal per year was US$ 5.69. However, effective tsetse control does not require the application of RAP to all animals. Protecting cattle from trypanosome infections by spraying 25 %, 50 % or 75 % of all cattle in a village costs US$ 1.72, 3.45 and 5.17 per animal per year respectively. Alternatively, a year of a single dose of curative or prophylactic trypanocide treatment plus 50 % RAP would cost US$ 4.87 and US$ 5.23 per animal per year. Pyrethroid insecticides and trypanocides cost 22.4 and 39.1 % of the cost of RAP and chemotherapy respectively.

**Conclusions:**

Cost analyses of low cost tsetse control options should include full delivery costs since they constitute 77.6 % of all project costs. The relatively low cost of RAP for AAT control and its collateral impact on tick control make it an attractive option for livestock management by smallholder livestock keepers.

## Background

African trypanosomes transmitted by tsetse flies (Diptera: *Glossinidae*) cause human African trypanosomiasis (HAT) and African animal trypanosomiasis (AAT), a debilitating disease of domestic animals and humans in the humid and sub-humid zones of Africa [1–4]. The tsetse-infested regions of Uganda include regions where livestock constitute the mainstay of livelihoods [5, 6]. Across much of this region, cattle are the main reservoir of human infective *Trypanosoma brucei (T.b) rhodesiense* [7–9]. Uganda is unique in that it has the human disease foci for both the chronic form of HAT caused by *T. b. gambiense* and the acute form, caused by *T.b rhodesiense* [9–12]. There is a serious risk of a merger of the two forms of disease as a result of cattle restocking following 20 years of unrest in the north and the north-eastern parts of the country [10, 11, 13–15]. This merger was arrested by a major intervention in 2006, the Stamp-Out Sleeping Sickness (SOS) project, whose objective was to remove infection from the major reservoir of infection in cattle by chemoprophylaxis and prevent reinfection by pyrethroid insecticide spraying of about 0.5 million cattle in six districts bordering Lake Kyoga [14, 16, 17].

Several tsetse and trypanosomiasis control methods have been developed and applied individually or in combination with varied levels of success in Uganda and elsewhere. These include those targeted at the vector: stationary baits [18–20], mobile baits (insecticide-treated cattle) [16, 21, 22], aerial spraying [23, 24], sterile insect technique (SIT) [25] and chemoprophylatic treatment of cattle [26]. Before deployment of AAT control methods, there is a need to evaluate both technical efficacy and the cost of the control method to be deployed so that informed choices can be made [27, 28].

In most African countries, veterinary services have been decentralised and privatised. Uganda is no exception and as a result, livestock keepers meet the costs of most of these services [29]. AAT control programs will need to tailor tsetse control to livestock keepers’ budgets to be sustainable [30]. The costs of different tsetse and AAT control options are needed to guide the process, aimed towards optimising resources [27, 28].

This study provides the first detailed economic costing of applying the restricted insecticide application protocol (RAP) for the control of tsetse and trypanosomiasis in Tororo District, south-eastern Uganda. RAP involves the application of pyrethroid insecticides on the tsetse predilection sites of the animal (the legs and bellies) [30]. In this study, deltamethrin (pyrethroid insecticide/acaracide), at dip concentration, was applied to the bellies, fore and hind legs, the preferred feeding sites for *G. f. fuscipes* and *G. pallidipes*. Deltamethrin was also applied to the ears of cattle, the usual predilection sites for *Rhipicephalus appendiculatus* ticks thus offering an additional collateral benefit of controlling *T.parva*, the infectious agent that causes East Coast Fever [31], which is a major constraint to livestock production and crop-livestock integration in this region [31–33]. The RAP costs provided here contribute data to guide tsetse and trypanosomiasis control programs. The values can be revised and contextualised to guide future large-scale use of RAP for AAT control programs.

## Methods

### Study area

This study was carried out in Tororo District, south-eastern Uganda (longitude 33.8–34.0; latitude 0.5–0.9) over a period of 18 months between June 2012 and December 2013. The district is bordered by the districts of Mbale to the north, Manafwa to the north-east, Busia to the south, Bugiri to the south-west, Butaleja to the north-west and the Republic of Kenya to the east. The district experiences two wet seasons, from September to November and from March to May. There are two dry seasons between June to August and December to February. Cattle are the main tsetse hosts in Tororo district [7, 8] contributing up to 54 % of all tsetse blood meals with the rest taken from pigs and monitor lizards (*Varanus niloticus*) [8]. *G. f. fuscipes* and *G. pallidipes* are the main tsetse fly species in the district [8]. There are localised foci of *G. pallidipes* especially in the north-eastern parts of Tororo District linked to re-introductions from Busia, Kenya [34]. Cattle in Tororo district play a key role in providing animal traction [31, 32].

### Cattle population included in the cost analyses

**Fig. 1:**
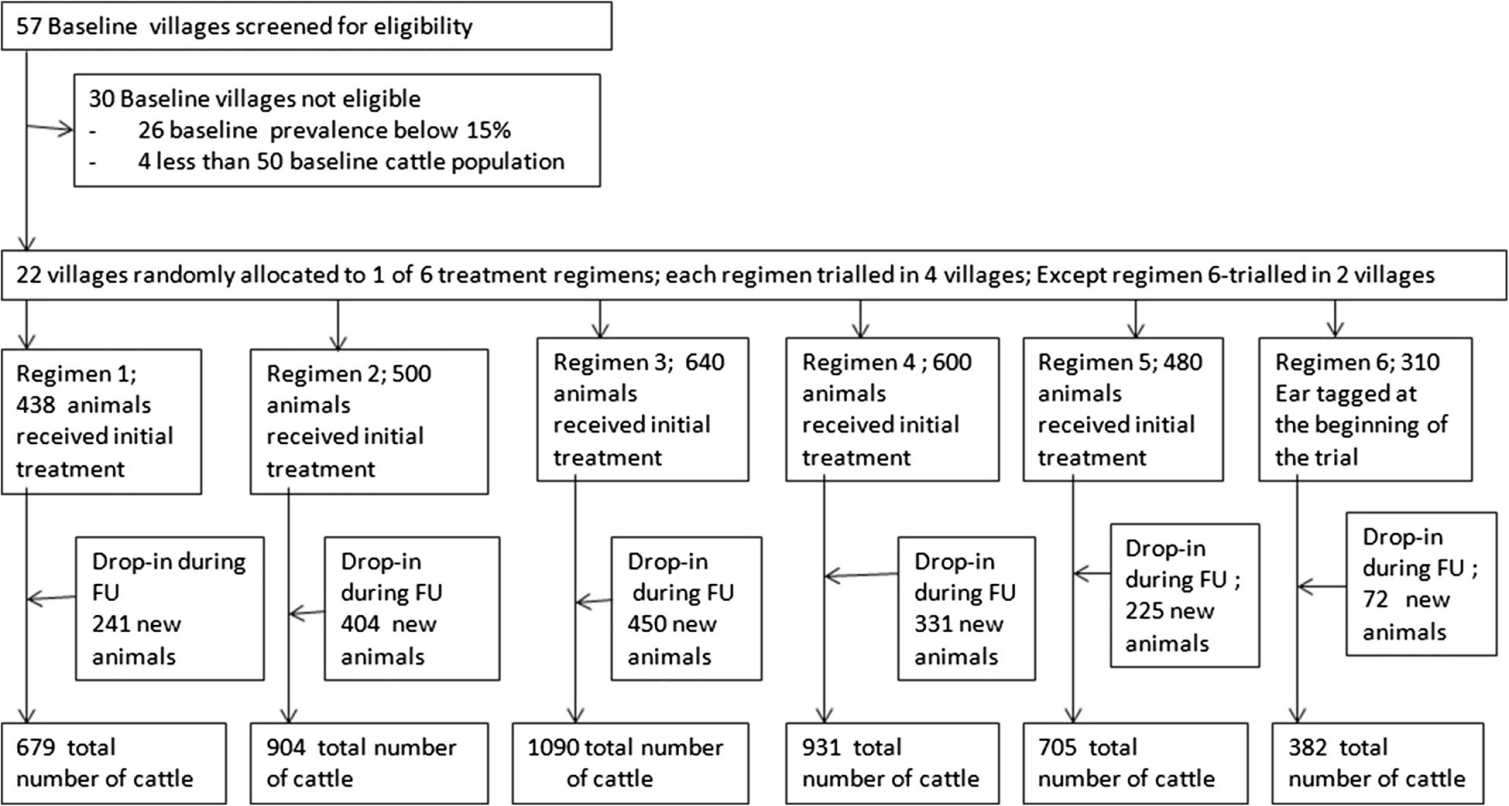
Study flow. Regimen 1: Diminazene diaceturate injections (DA); (0.01 g/kg body weight) forty days apart at the beginning of the trial. Regimen 2: DA and 25 % RAP. Regimen 3: DA and 50 % RAP. Regimen 4: DA and 75 % RAP. Regimen 5: DA and Albendazole 10 % drench (8 mg/kg body weight) - 3 monthly for 18 months. Regimen 6: No treatment at all. Median time of follow up-FU (time difference between first and last sampling of individual animals) was 12 months in each of the 6 treatment groups. The number in each of the regimens was arrived at by taking the average (taking care of births, deaths, purchases and sales) number of cattle present in the regimen per month for the 18 month intervention period

This study was based on a recent trial carried out to optimise the restricted pyrethroid insecticide application for simultaneous tsetse and tick-borne disease control [31, 32, 35, 36]. Of the 22 intervention villages selected from the 57 villages [31, 33], 12 villages were used to optimise RAP while the rest of the 10 villages were non-RAP regimens (Fig. 1). An accumulated 1469 cattle were sprayed in regimen 2, 3 and 4 representing 226, 545 and 698 cattle in each of the three RAP regimens respectively. All animals in the 22 intervention villages were tagged on introduction into the trial. To remove trypanosomes already present from study cattle so as to monitor the rate of reinfection during the 18 months of the study, all cattle over 3 months of age (except for those in 2 villages in regimen 6) were treated with Veriben B12 (Ceva Santé Animale, France) - a trypanocide containing diminazene diaceturate, cyanocobalamin (vitamin B12) and hydroxocobalamin (vitamin B12a) at a dose of 0.01 g/kg live body weight by deep intramuscular injection. Details of livestock keepers, their household co-ordinates (village, parish, county) and their cattle demographics (age, sex, breed,) were entered in a herd structure register on introduction into the intervention study, which was updated every three months [31, 36].

RAP was applied in twelve villages; three groups of four villages that were allocated different regimes for spraying fixed proportions (25, 50 and 75 %) of the total village cattle herd [36]. An emulsifiable Deltamethrin concentrate (Vectocid®, Ceva Interchem, Tunis) spray was applied at dip concentration of 0.1 % deltamethrin (1:1000; Vectocid to water parts) on the legs, bellies and ears of cattle, every 28 days over 18 months. Three control groups were included in the study; 2 villages where no interventions were undertaken; 4 villages where cattle were only given two injections of a trypanocide forty days apart at the beginning of the project and 4 villages in which cattle were dewormed once every three months. In all the villages (except those where no treatment was administered) blood samples were taken fourteen days after the second Veriben B12® treatment, repeated at three monthly intervals to monitor trypanosome and *T.parva* prevalences. In the two “no treatment villages” blood was sampled on initial cattle tagging and at 6 and 12 months. All cattle in the ten non-RAP villages were treated against trypanosomiasis on termination of the intervention either at 12 or 18 months, for ethical reasons since they were at higher risk of acquiring AAT.

### Inputs

Project implementation required inputs both at livestock keeper and project levels. Inputs from the livestock keepers included: movement of cattle to the cattle treatment centres, provision of water for spraying, cattle handling (e.g. ropes, crushes and physical labour) and community mobilisation (by community heads or livestock keepers themselves). Inputs from the project intervention side included Albafas® (Albendazole 10 %, Norbrook Laboratories Ltd, Kenya), Vectocid® and Veriben B12® bought in bulk from suppliers in Kampala. Major capital expenses included acquiring and servicing equipment like; spray pumps, a Hilux pick-up vehicle, GPS units and a laptop computer. Consumables and re-usable items included; ear-tag applicators and ear tags, 20 ml syringes for trypanocidal administration, needles, syringes for de-wormer administration (50 ml), sterile water for injection, protective clothing (examination gloves, gum boots, face masks and overalls), assessment tools such as Flinders Technology Associates (FTA) cards, silica gel desiccants, capillary tubes, lancets, FTA card pouches and office stationery. Communication, vehicle (fuel, servicing and repairs), staff salaries and allowances formed the bulk of recurrent expenses. For analysis, these expenditures were categorised as capital or recurrent expenditure. All inputs that were used in monitoring of trypanosome and *T.parva* prevalence, cattle deworming, maintenance of the herd structure register and principal investigators’ salaries are associated with the research component of the project and were treated independently in order to arrive at the costs attributable to the control of trypanosomiasis (RAP with and/or without curative trypanocide administration).

### Economic analyses

The total or full cost calculation approach [27] was used to undertake an economic rather than a financial analysis. In an organisational setting, initiating this type of operation would require additional expenditure for veterinary pharmaceuticals, taking of blood samples, staff travel allowances and vehicle running costs. These variable costs, often referred to as ‘direct’ are often the only ones reported. A share of various overheads (fixed costs) such as depreciation on capital items and a share of staff salaries was added to these costs to calculate the full delivery cost. Cost calculations were undertaken using Microsoft Excel^TM^ spreadsheets, without incorporating probabilistic elements, since costs related to tsetse control programs vary with habitats, tsetse species, and organisational contexts rather than following a probability distribution [27]. The analysis covered the 18 months of the project to provide fair estimates of the value of all inputs over that period, including those shared with other activities and a share of any relevant overheads. Depreciation on all capital items used was calculated over the 18 month period using the straight line method, based on estimated useful life and likely salvage value at the end of that period, where relevant. To calculate the economic (societal) cost of the intervention, transfer payments (taxes) were netted out. Apart from this, market prices were used without adjusting for externalities and /or market distortions [27]. Opportunity costs of cattle-keepers’ time were valued using appropriate proxies, in this case the payment of a modest amount of US$ 3.9 per village visit to the village head who would in turn buy refreshments for the cattle owners and recover his community mobilisation costs. A further study, based on livestock keeper interviews, is investigating the cost to livestock keepers. All costs are based on expenditure in Uganda Shillings (UGX) or US$ at 2012/2013 prices. All UGX are converted to US$ at the rate of UGX 2575 = 1 US$, which applies to the study period

(http://www.oanda.com/currency/historical-rates-classic).

Analyses were performed at three levels: i) full costs of all the project activities indicating a share that was spent on research, ii) costs of Veriben B12® injections, and monthly RAP for 18 months and iii) costs of RAP only. Costs of RAP delivery were calculated based on the number (1469) of cattle sprayed. The costs of delivering each group of activities were expressed per animal per month (28 days). Direct costs including vehicle running costs, Veriben B12® and Vectocid® were expressed as a percentage of the overall total costs in each of the three categories.

### Ethical clearance

This study was reviewed by the Makerere University College of Veterinary Medicine Animal Resources and Biosecurity ethical review board for compliance to Animal use and Care Standards. It was then forwarded to the Uganda National Council for Science and Technology and approved under approval number HS1336.

## Results

### Costs of all project activities including biophysical monitoring (research) for 18 months of follow-up

This component covers the full delivery costs from the project side. These costs included those primarily related to AAT control, namely application of RAP and Veriben B12®. It also provides a detailed costing of activities for monitoring project effectiveness (*Trypanosoma spp*. and *T.parva* prevalences) that were not necessary for implementing RAP or administering trypanocidal drugs. These included blood sampling, cattle ear-tagging, deworming, livestock keeper registration, updating the herd structure register and a share of community mobilisation costs. The average number of animals present in all 22 villages over the 18 month intervention period was 4,691 head. The average number of animals in the 20 villages where RAP, chemotherapy and deworming were undertaken was 4,309 head of which 1,469 in 12 of the 20 villages received monthly RAP. In addition, 705 head in four villages were dewormed once three-monthly for 18 months (Fig. 1). A summary of the costs of the overall project over the 18-month intervention period clearly indicating the cost that was spent on research activities is presented in Table 1. Direct research related activities constituted 55 % of all the project costs.

**Table 1:** Full cost of RAP, Veriben B12® injections, deworming and biophysical monitoring (research) for 18 months

Cost items	Intervention cost = X (US$)	Research cost = Y (US$)	Total cost = Z (US$)	X as % of total^a^	Y as % of total^a^	Z as % of total^a^
A) Capital items						
Specialised equipment (spray pumps)	583	0	583	0.4	0.0	0.4
Vehicle	2,200	300	2,500	1.4	0.2	1.6
Other equipment (laptop and GPS units)	66	0	66	0.0	0.0	0.0
Total capital	2,848	300	3,148	1.8	0.2	2.0
B) Recurrent expenditure						
Spray pump service	450	0	450	0.3	0.0	0.3
Vectocid	3,402	0	3,402	2.1	0.0	2.1
Cattle ear tags and ear tag applicators	0	5,583	5,583	0.0	3.5	3.5
Protective gear (gloves, overalls, masks and gumboots)	362	314	677	0.2	0.2	0.4
Trypanocidal injections (Veriben, needles, syringes, injection water)	3,477	0	3,477	2.2	0.0	2.2
Albendazole 10 % and its administration syringes	1,437	0	1,437	0.9	0.0	0.9
Sample taking, packaging and postage (capillary tubes, lancets, FTA cards and pouches, silica gel desiccants and courier fees)	0	15,682	15,682	0.0	9.8	9.8
Stationery	0	1,400	1,400	0.0	0.9	0.9
Vehicle running and travel	8,758	1,194	9,953	5.5	0.8	6.2
Staff salaries	25,920	43,080	69,000	16.2	26.9	43.1
Staff travel allowances	24,418	17,444	41,862	15.3	10.9	26.2
Payments to village heads and communication	1,049	2,911	3,959	0.7	1.8	2.5
Total recurrent	69,273	87,609	156,881	43.3	54.8	98.0
C) Overall total	72,121	87,909	160,029^a^	45.1	54.9	100.0
Project cost per animal for 18 months (n:4,309)	16.7	20.4	37.1	45.1^b^	54.9^b^	100.0 ^b^
Cost per animal per month	0.93	1.13	2.06	45.1^c^	54.9^c^	100.0^c^

^a^Total Project Cost,^b^as a percentage of animal cost for 1.5 years, ^c^as a percentage of animal cost per month

### Costs of implementing monthly RAP for 18 months and initial Veriben B12® injections

Four thousand, three hundred and nine (4309) cattle in 20 villages were injected with Veriben B12® intramuscularly on introduction into the trial. This constituted a major cost of this program implementation. An average of 1,469 of the 4,309 cattle in 12 of the 20 villages was sprayed, using RAP, once every 28 days. Table 2 presents a summary of the costs of delivering RAP and Veriben B12® intramuscular injections at the start of the trial.

**Table 2:** Costs of implementing monthly RAP for 18 months and initial Veriben B12® injections

Cost items	Total (US$)	% of the Total
A) Capital items		
Specialised equipment (spray pumps)	583	1.0
Vehicle	1,900	3.2
Other equipment (laptop and GPS units)	65	0.1
Total capital	2,548	4.3
B) Recurrent expenditure		
Trypanocidal injections (Veriben B12, needles, syringes, injection water)	3,476	5.9
Vectocid	3,402	5.8
Spray pump service	450	0.8
Protective clothing (gloves, facemasks, overalls, gumboots)	516	0.9
Payments to village heads and communication	912	1.5
Vehicle running and travel	7,564	12.8
Staff salaries	20,700	35.0
Staff travel allowances	19,519	33.0
Total recurrent	56,539	95.7
C) Overall total	59,087	100.0
RAP &Veriben B12 per individual animal for 18 months (n:4,309)	13.71	–
Cost per animal per month	0.76	–

### Costs of delivering monthly RAP for 18 months

This section presents the cost of applying RAP to 1,469 cattle in 12 villages of Tororo District for 18 months at intervals of 28 days. The total number sprayed over time includes cattle introductions accruing from purchases, exchanges, gifts and births. This required three animal health providers (one veterinary surgeon and two livestock health assistants) working on average 8 days in a month for 18 months to undertake community mobilisation or spraying of cattle. Table 3 shows the direct costs, planning and organisational overheads that were incurred to maintain RAP for 18 months.

**Table 3:** Costs of implementing monthly RAP for 18 months

Cost items	Total (US$)	% Total costs
A) Capital items		
Specialised equipment (spray pumps)	583	3.8
Vehicle	800	5.3
Other equipment (laptop and GPS units)	65	0.4
Total	1,448	9.5
B) Recurrent expenditure		
Spray pump service	450	3.0
Vectocid	3,402	22.4
Protective clothing for staff (gumboots, overalls, gloves and facemasks)	241	1.6
Payment to village heads and communication	295	1.9
Vehicle running and travel	3,185	21.0
Staff salaries	4,522	29.8
Staff travel allowances	1,648	10.8
Total recurrent	13,743	90.5
C) Overall total	15,191	100.0
Cost per individual animal for 18 months (n: 1,469)	10.34	–
Cost per animal per month	0.57	–

### The annual cost of protecting cattle from trypanosome infections using graded (25, 50 and 75 %) RAP

The unit cost of protecting cattle with graded RAP is much lower than the unit cost of RAP per animal sprayed. For example in the 4 villages containing 914 head of cattle only 229 cattle were sprayed to achieve a 25 % RAP coverage. This indicates that the annual unit cost of protecting 914 cattle from trypanosome infections in the 25 % RAP coverage is US$ 1.72. This cost increases to US$ 3.45 and 5.17 in the 50 % and 75 % RAP coverage villages respectively (Table 4). These costs per regimen and their average (US$ 3.45) are actually lower than the cost of RAP per animal sprayed for 18 months at US$ 10.34 (Table 3) or the equivalent of US$ 6.89 per animal per year.

**Table 4:** The annual cost (US $) of protecting a cattle population by spraying different proportions of the cattle population using RAP

RAP coverage	Cattle (n)	Average sprayed	Total RAP cost	Annual cost
25 %	914	229	1,574.37	1.72
50 %	1,096	548	3,775.72	3.45
75 %	931	698	4,810.94	5.17

## Discussion

This study analysed the costs incurred for applying RAP to 1,409 cattle in 12 villages in Tororo District to assess the applicability of RAP for AAT management in small-scale livestock production systems. Trypanocidal chemotherapy and RAP were targeted at AAT control. Non-tsetse control activities related to monitoring project effectiveness (Trypanosome species and *T.parva* prevalences) namely: blood sampling, herd structure records and deworming, comprised 55 % of the costs. Such research costs, together with administrative overheads often account for a high proportion of the expenditure for AAT control and vary greatly from strategy to strategy but are often omitted from published cost figures for interventions [37]. For technologies described as lower cost technologies such as RAP, a substantial share of the costs has previously [27] been reported and confirmed here (77.6 % of all costs) to be due to delivery. This cost is divided between inputs by the veterinary or health services of a specific project and the often, unquantified, inputs by livestock keepers themselves. For this reason, the costs calculated here for delivering RAP and preventive chemotherapy together or separately are slightly higher than most of those previously reported.

Only a few studies on application of pour-on for tsetse and trypanosomiasis control have attempted to include both direct and indirect costs [38, 39]. Even then, these studies looked at the use of pour-on which is often too expensive for small-holder livestock keepers. Application of pyrethroids using the pour-on methodology has been reported to use 10 times the amount of insecticide/acaracide compared to RAP at dip concentration and risks high pyrethroid environmental exposure [30, 40–42].

In this study, the full cost of applying pyrethroid insecticides to the legs, bellies and ears is US$ 0.57 per animal per spray with the cost of the pyrethroid (in this case Vectocid) representing 22.4 % (US$ 0.13) of all costs. This cost is about twice the cost of US$ 0.25 (UK£ 0.14) cited for 2005 in an earlier study [43]. That study estimated that including delivery would approximately increase total cost to tenfold that of the insecticide, comparable to the figure obtained here from detailed recording of full costs. In that study an α-cypermethrin formulation was used, which is generally cheaper than the deltamethrin formulation used in this work to which inflation (30 %) for Uganda during the period 2005–2013 would need to be added. In this study, unlike those in the past [30, 43], the ears of cattle were sprayed to add an additional benefit in the form of *T.parva* control which further explains the higher cost figure obtained.

To estimate the current cost of delivery of a single curative dose of Veriben B12®, the delivery costs per animal per month, excluding research supervision and all costs related to deworming, spraying and injecting were calculated using the data presented in Tables 1, 2 and 3 as US$ 0.79, 0.64 and 0.39 respectively. The average of these three figures is US$ 0.61, which represents the full cost of delivering an intervention using the same delivery structure as was developed for RAP. The cost of Veriben B12® came to US$ 0.64 per dose (of 192 kg body weight) and the cost of needles, syringes, sterile water came to US$ 0.17 per animal. Thus the total cost for delivery of Veriben B12® was US$ 0.61and the total cost per injection of Veriben was US$ 1.42. The cost of Isometamidium chloride (Samorin®) is higher, at about US$ 1.56 per 300 kg body weight dose, so approximately US$ 1.00 per 192 kg bodyweight animal, which would come to US$ 1.78, if delivery cost were added.

Comparing the cost to farmers of protecting their animals from trypanosomiasis using RAP or trypanocides is not straightforward. As such, the two are not mutually exclusive depending on the disease epidemiology in a given area. In Tororo district for example, *T. b rhodesiense* transmission rate was recently reported to be very low with a district prevalence of about 0.03 % [35, 36]. On the other hand this area continues to have a significant level of *T.congolense* and *T.vivax* transmission rates which are rather hard to control compared to *T. b rhodesiense* [35, 36]. Since AAT due to *T.vivax* and *T.congolense* is still a major constraint to livestock production in this area [35, 36], the proportion of cattle to be sprayed would need to be increased to 50 % with a once-off curative or prophylactic trypanocidal treatment per year for 2–3 years to leverage AAT control [36, 44, 45]. Since tsetse flies are more attracted to larger and older (often more productive animals) animals in the herd than small and younger animals [46], this category of cattle would constitute the proportion of cattle to be sprayed. To leverage control of AAT due to *T.congolense* and *T.vivax* whose population decay as a result of RAP alone is slow [44, 45], all cattle in the district would in addition need to be treated once per year with either a curative or prophylactic trypanocide [36]. Trypanocidal treatments would then be repeated for 2–3 years to provide a sufficient HAT and AAT control regime. Curative and prophylactic treatments would need to be alternated as a sanative pair between years 2–3 in order to prevent the likelihood of development of drug resistance against either chemical [26, 47].

Integrating RAP usage with strategic trypanocide administration explained above reduces over-dependence on trypanocides; lowers the risk of drug resistance and the cost of tsetse and tick-borne disease control with a proportionate reduction in the risk of environmental damage [31, 36, 48]. For example, Tororo District had about 37,000 cattle at the time of this study. To maintain all cattle in the district on RAP for a year, US$ 254,930 would be needed. If the interest was to control acute HAT, only 25 % RAP coverage would be sufficient. The unit cost of protecting cattle from trypanosome infections would then be US$ 1.75 per animal per year and would cost a quarter to half of the above budget; US$ 63,733. A year of curative trypanocidal treatment and 50 % RAP coverage would cost the farmers in the district US$ 180,005 or US$ 4.87 per bovine while that of prophylactic trypanocidal treatment and RAP would cost US$ 193,325 or US$ 5.23 per bovine.

To protect cattle from trypanosome infections using 25, 50 and 75 % RAP coverages alone would cost US$ 1.72, 3.45 and 5.17 per animal present per year (Table 4) respectively which is much lower than the cost of maintaining each animal on RAP per year (US$ 6.89). This is because it has earlier been reported that spraying just a proportion of a village cattle herd is sufficient for tsetse and trypanosomiasis control [30, 36]. It is for this reason that we ought to differentiate between the average cost of RAP per animal sprayed per year and the average cost of protecting cattle from trypanosome infections using different RAP herd coverage levels. It is here reported that the latter cost is indeed much lower than the former.

Trypanocide application is the most popular approach for trypanosomiasis control by livestock keepers in the tsetse infested areas of Africa [26, 30, 49, 50]. Some 35 million doses were used in 2004 costing about US$ 30 million to the livestock industry [26]. Trypanocide use is popular with livestock keepers because it is believed by farmers to provide rapid, private and inexpensive means of trypanosomiasis control compared to tsetse control methods [30]. If farmers were to protect their cattle by using trypanocides only, they would need to administer some four or two doses of curative or prophylactic trypanocides respectively, per animal per year. This is due to the short window of protection afforded by curative trypanocidal treatments compared to prophylactic trypanocides [41]. As costed above, curative and prophylactic trypanocide treatments would cost them about US$ 5.69 (four doses) and US$ 3.57 (2 doses) per animal per year respectively. However, extensive use of trypanocides is threatened with development of drug resistance [26, 47]. Moreover, cattle that are produced under extensive trypanocide use are less productive than those kept under low tsetse challenge by use of insecticides [41, 44, 45]. In addition, the cost of chemotherapy is highly variable depending on whether trypanocidal drugs are bought in bulk or in small amounts, the expertise of the veterinary service provider, and the distance from point of veterinary care. Smallholder livestock keepers in Africa for example, keep a few cattle (an average of 3 in Tororo) and procure trypanocides in small quantities at a higher price than project drugs would be purchased in bulk from large wholesale urban traders [41]. In this study, veterinary treatment costs were shared by very many livestock keepers and spread over a sizeable cattle population (4,309 head) indicating that the cost to the farmer in Tororo could have been higher.

The costs explained above should therefore be seen in relation to the impact on the prevalences of *Trypanosoma spp*. and *T. parva.* RAP is likely to maintain a small population of ticks on cattle that maintains a small force of infection in the cattle population [30, 31]. Consequently, cattle are exposed to a small population of ticks and therefore to tick-borne infections. This is likely to promote and maintain endemic stability in the cattle population, a process by which a large proportion of the herd is exposed to tick-borne infections as calves and remain with a solid immunity against similar infections as adults [31, 48, 51]. In addition, RAP being a relatively low cost technology with additional impact on TBDs together with the fact that it is environmentally benign, have all been reported as some of the collateral benefits of this technology [30, 40, 42, 52]. For these reasons, it has previously been suggested [30, 40, 41] and is recommended here, that RAP be adopted for simultaneous tsetse and tick-borne disease control in small holder crop-livestock production systems.

For many AAT control programs, the bulk of the intervention costs are for delivery and these need to be fully quantified and included in future costing. In addition costs related to project monitoring can also be substantial and need to be teased apart from the costs of implementing AAT control programs and quantified. Of all the costs needed for this project’s implementation, 55 % were related to project monitoring.

## Conclusions

This study showed that RAP could be delivered at US$ 6.89 per animal per year which is comparable to the cost of the delivery of four doses of curative trypanocide at US$ 6.04 needed to keep cattle under high AAT transmission areas like Tororo district. However, effective tsetse control does not require the application of RAP to all animals. Protecting cattle from trypanosome infections with 25 %, 50 % or 70 % RAP herd coverage would, for example, cost US$ 1.72, 3.45 and 5.17 per head per year respectively. Alternatively, a year of a single dose of curative or prophylactic trypanocide treatment plus 50 % RAP would cost US$ 4.87 and US$ 5.23 per animal per year. Integrating strategic trypanocide usage and RAP therefore provides cheaper and safer (reduced risk of drug resistance and damage to non target organisms) means for tsetse and trypanosomiasis control. For both RAP and chemotherapy 77.6 and 60.9 % of costs were for delivery; thus it is recommended that future cost analyses, especially of low cost techniques, include a full share of all overheads involved in delivery and project preparation. The relatively low cost of RAP (US$ 0.57 per animal per spray; US$ 6.89 per animal per year) for AAT control and its collateral impact on tick control make it an attractive option for livestock management by smallholder cattle keepers.
